# Detecting Helical Gearbox Defects from Raw Vibration Signal Using Convolutional Neural Networks

**DOI:** 10.3390/s23218769

**Published:** 2023-10-27

**Authors:** Iulian Lupea, Mihaiela Lupea

**Affiliations:** 1 Faculty of Industrial Engineering, Robotics and Production Management, Technical University of Cluj-Napoca, 400641 Cluj-Napoca, Romania; 2 Faculty of Mathematics and Computer Science, Babes-Bolyai University, 400084 Cluj-Napoca, Romania; mihaela.lupea@ubbcluj.ro

**Keywords:** helical gear fault detection, accelerometer sensor, convolutional neural network, vibration signal

## Abstract

A study on the gearbox (speed reducer) defect detection models built from the raw vibration signal measured by a triaxial accelerometer and based on convolutional neural networks (CNNs) is presented. Gear faults such as localized pitting, localized wear on helical pinion tooth flanks, and lubricant low level are under observation for three rotating velocities of the actuator and three load levels at the reducer output. A deep learning approach, based on 1D-CNN or 2D-CNN, is employed to extract from the vibration image significant signal features that are used further to identify one of the four states (one normal and three defects) of the system, regardless of the selected load level or the speed. The best-performing 1D-CNN-based detection model, with a testing accuracy of 98.91%, was trained on the signals measured on the Y axis along the reducer input shaft direction. The vibration data acquired from the X and Z axes of the accelerometer proved to be less relevant in discriminating the states of the gearbox, the corresponding 1D-CNN-based models achieving 97.15% and 97% testing accuracy. The 2D-CNN-based model, built using the data from all three accelerometer axes, detects the state of the gearbox with an accuracy of 99.63%.

## 1. Introduction

Smart manufacturing associated with Industry 4.0 requires intelligent methods to detect faults in gears, bearings, and other machine elements. Traditionally machine elements fault diagnosis methods need professional knowledge of the human operator and qualified interpretation of the acquired sensor data, especially vibration signals. A gear pair is a significant subassembly in machines for rotation and power transmission from one shaft to another. Gears play a vital role in transportation and industrial manufacturing applications, including gearboxes and speed reducers. Gears are expected to work continuously for a long time in the production system; hence, the demand is for high reliability, increased load-carrying capacity, and endurance life, in parallel with lower vibrations and noise. Faults on gears may cause unexpected machine breakdowns associated with significant economic loss and sometimes personnel casualties. The complex feature extraction procedures, mainly from acceleration sensors, participate in classification by using pattern recognition models. Early fault diagnosis of the gears may prevent failures of the system. The fatigue of the materials on contacting surfaces in gears is often the life-limiting factor in gears transmission. In that regard, the teeth flank surfaces require grinding, super finishing, or other surface treatments.

**Figure 1:**
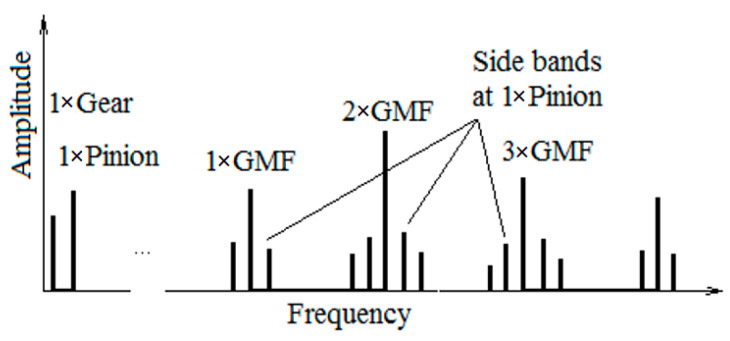
GMF and sidebands on pinion.

Once a machine fault develops, the machine condition with respect to the vibration profile changes. The generated noise, temperature, and lubrication conditions change as well. The normal vibrations from the gear pair are mainly produced by the shocks between the teeth as the gears mate during operation. The vibration that is monitored on a healthy gear exhibits a significant level of vibration at the tooth meshing frequency (GMF), which is calculated by multiplying the number of teeth of the gear wheel or pinion with its rotational speed. Important components in gear vibration spectra are at the GHF harmonics as well. The acquisition system has to observe a frequency bandwidth of about 10 kHz or more. A faulty gear exhibits sidebands on both sides of the GMF and harmonics at a distance on the frequency axis that equals the rotational speed of the faulty wheel (Figure 1).

Often, a gearbox presents multiple gear pairs. The vibration spectrum of each gear pair contains important frequency bands at the GMFs and associated sidebands caused by modulation of a carrier signal (GMF-center frequency) by a modulating signal (pinion rotational speed). Sidebands are also present at the harmonics of each gear mesh frequency. The more energy there is in the sidebands, the amplitude levels of the sidebands will increase, meaning that the damage in gear teeth becomes larger [1,2,3,4,5]. Typical faults (defects) on gears are abrasive wear, adhesive wear, micro-pitting, pitting, chipping, scuffing (a form of adhesive wearing), spalling, crack, tooth breakage, and white structure flaking [6]. These are found to initiate the majority of the gearbox failures. Some of the faults, like Hertzian fatigue (pitting and micro-pitting), wear, and scuffing/scoring, are associated with lubrication problems. Faults resulting from overloading and fatigue due to bending at the tooth root (root fillet crack), plastic flow, or tooth fracture [6] are non-lubrication-related failures.

Micro-pitting is a cluster of micro pits, also known as peeling, superficial spalling, or grey staining [7]. Micro-pitting means pits smaller than 10 microns in size that occur under poor elastohydrodynamic lubrication conditions. Micro-pitting may lead to vibrations, noises, and misalignments or even contribute to the appearance of pitting, spalling, scuffing, and tooth breakage.

Pitting is characterized by the detachment of metal fragments, visible to the naked eye, as a consequence of Hertzian fatigue, one of the most common faults in gear teeth. It starts from fatigue cracks initiated on the tooth flank surface or at a small depth under the flank surface. Above and below the pitch line on the flank, pitting is more likely to appear because the sliding of the flank opposite surfaces is added to the pure rolling.

Gear pitting and wear fault detection can follow model-based methods by observing changes in system dynamics. Traditionally, gear faults are identified through vibration signal processing techniques such as those in the time domain, frequency domain, and time-frequency domain.

Numerous condition indicators are statistical parameters derived from the time domain signal. Some of them indicate the central tendency of the vibration signal (root mean square, mean absolute value), some indicate dispersion (standard deviation, variance), distribution anomalies (such as skewness, kurtosis, and high-order moments), or the shape of waveforms like crest factor.

Among time-averaging methods, a powerful tool in the signal conditioning and detection of gear faults is the time synchronous average (TSA), which considerably reduces the background noise and periodic events that are not synchronous with the gear of interest [8]. Accelerometer and tachometer sensors are required to average blocks of vibration data representing one revolution of the observed gear. The TSA often precedes the calculation of gear status indicators.

Plenty of parameters (condition indicators) in the time domain were derived (from TSA, regular, difference, or residual signals) to catch faults. The zero-order figure of merit (FM0) is a coarse fault detection parameter indicating major faults in a gear mesh by detecting important changes in the meshing pattern, like tooth breakage or heavily distributed wear. The FM0 indicates well gear tooth pitting present in several teeth. The fourth-order figure of merit (FM4) recognizes damage restricted to a few (one or two isolated) gear teeth, losing the sensitivity as the damage spreads to other teeth [9]. NA4 detects the onset of damage, as FM4 does, and also continues to react to gear tooth surface damage like isolated pitting as it spreads and grows in severity [9]. M6A and M8A are variations of the sixth (M6) and eighth (M8) normalized statistical moments and are used to detect surface damage using vibration signals. These parameters present stronger reactions to the damage as compared to FM4 [9]. NB4 indicates localized gear faults on a few teeth that cause load fluctuations (observed on the envelope of the signal, computed by using the Hilbert transform), as opposed to load fluctuations caused by healthy teeth [5]. In general, a couple of parameters need to cooperate in order to predict pitting.

For stationary signals, the fast Fourier transform (FFT) is the basis of the frequency analysis and the derivation of the frequency features. Some indicators in the frequency domain are the mean frequency, root mean square frequency, standard deviation frequency, spectral centroid, spectral spread, spectral skewness, spectral kurtosis, and so forth. These statistical parameters are often employed for fault detection using supervised machine learning techniques [10]. One can add special spectral coefficients like GMF, GMF harmonics, and their associated side bands. Based on the sideband energy of the GMF and multiples, the sideband ratios, sideband level factors, and sideband index were defined [10] to account for the wear damage on gear flanks and other faults. A sideband ratio for the first two meshing harmonics has been used to differentiate gear pitting from healthy conditions [11].

Vibration data can be accompanied by acoustic signals acquired from microphones. In [12], a pure acoustic analysis based on the gears’ sound is mentioned. The spectral data of a few specific frequency bands of the recorded sound are used as input for a deep-learning model in order to diagnose the defects in gears.

For a machine with varying speed or load, such as, for example, in wind turbine transmissions, the system dynamics and the measured vibrations are non-stationary. When a fixed time sampling for the whole signal is used and the rotational frequency of the machine varies, the result is increased leakage error and spectral smearing. Time-frequency approaches applied for non-stationary signal processing are the short-time Fourier transform (STFT), wavelet transform (WT), Wigner–Ville distribution (WVD), Hilbert–Huang transform (HHT), and the order tracking technique. In the STFT, the FFT is applied repeatedly to a short time window of the signal with a time step of the moving window, resulting in a spectrogram of the signal (power spectrum variability with time). A good frequency resolution and a poor time resolution are achieved for a larger time window and vice versa (for a shorter time window); hence, both good time and frequency resolutions cannot be obtained. A special case is the Gabor transform, when one uses the Gaussian function or Gabor function for the window [13].

In [14], a single gear condition indicator, the sideband power factor (SBPF), was proposed to evaluate the gear damage for the non-stationary load and speed operating conditions of a wind turbine (tested for a two-stage helical gear parallel shaft gearbox). The power spectrum amplitude of the second harmonic of the meshing frequency and its first five sideband peaks on each side are summed together; the sidebands are associated with the pinion turning speed. Wear on the leading contact edge of a tooth is observed; progressive damage in three stages is artificially introduced from the early stages up to a 7 mm × 5 mm missing chip.

Deep learning has contributed significantly to automatic fault diagnosis by extracting directly from the raw vibration signal the most important features that detect defects.

A convolutional neural network (CNN), a deep learning technique, is an extension of an artificial neural network (ANN) used to extract features from grid-like matrix datasets, such as images. This type of network learns abstract features by alternating and stacking convolutional layers and pooling layers. The convolutional layers convolve multiple local filters with raw input data and generate translation-invariant local features. The subsequent pooling layers extract features with a fixed length by sliding windows over the input data and applying *average* or *max* functions.

In general, a CNN processes images; hence, the applications in machinery health monitoring are based on 2D data generated by STFT or continuous wavelet transform (CWT), starting from the vibration signal. CNNs also proved to achieve good results for 1D time-series data. A CNN digs and extracts the deep features in the images related to fault vibration and then classifies the vibration data.

A study on the gearbox defect detection models built from the raw vibration signals measured by a triaxial accelerometer and based on convolutional neural networks (CNNs) is presented in this paper. We will follow a data-driven method by observing the changes in the measured vibration data for two pinion defects: a small pitting one about the pitch line on a flank and localized wear at the tooth addendum. The artificial defects were made using micro diamond burs. A total of four health states were under observation by adding a lubrication state and a healthy, normal state. The experiments are carried out for three different speeds and three load conditions. CNN-based models are used to learn deep features from the vibration images; features that discriminate between the four states of the gearbox.

The research goals proposed in this paper can be summarized as follows.

RG1. Proposing and implementing a methodology that solves the defect detection problem for a gearbox (four health states), regardless of the selected load level or speed on the test rig, using deep learning CNN-based models built from raw vibration signals measured by a triaxial accelerometer.

RG2. Performing a comparison between the performances of the defect classification models built from the vibration information extracted from individual accelerometer axes (X, Y, and Z) and using 1D-CNN. The aspect of identifying the axis most closely associated with each specific defect will also be addressed.

RG3. Developing a defect detection model based on the 2D image representation of the raw vibration signal measured on all three axes and using 2D-CNN.

The rest of the paper is organized as follows. Related work that approaches the gearbox defect detection problem is presented in Section 2. Section 3 is dedicated to presenting the test rig used in the experiments and describing the gearbox defects under observation in the study. The first two steps of the proposed methodology, i.e., the data acquisition and processing, are described in Section 4. The proposed 1D-CNN and 2D-CNN-based classification models designed to detect the four states (classes) of the gearbox are discussed in Section 5. The experiments and the evaluation of the defect detection models are presented in Section 6. The Section 7 contains the conclusions of the present study.

## 2. Related Work

In [15], gear defects on variable speed machinery are detected by using a method that combines computed order tracking, cepstrum analysis, and a radial basis function (RBF)—two-layer artificial neural network (ANN) type. The gearbox (one stage) signal amplitude from constant times is resampled at constant rotational angles, then transformed in the order domain using FFT and processed by cepstrum analysis. Order spectrum gives the amplitude of the signal as a function of the harmonic order and shaft speed in rotating machinery. A crack on the root of a spur gear tooth and severe localized wear on the driving gear tooth was artificially made by an electric discharge machine and successfully detected with the RBF-ANN.

The concept of time-frequency entropy based on the Hilbert–Huang transform (HHT) is proposed for gear fault diagnosis in [16]. HHT includes empirical mode decomposition (EMD), decomposing a complicated signal into several intrinsic mode functions (IMFs). HHT can be applied to non-linear and non-stationary signals. The concept offers accurate energy–frequency–time distributions in the measured vibration signal, classifying the gear as without fault (energy in GMF and multiples) and with a fault (extra energy in sidebands). A tooth crack fault and a broken tooth have been considered, but the method could not distinguish between faults.

In the last two decades, traditional fault detection methods have been gradually replaced by methods based on artificial neural networks with good learning and classification capabilities [17]. The main deep learning directions in machine health monitoring systems are autoencoders (and variants), restricted Boltzmann machines (and variants), convolutional neural networks (CNNs), and recurrent neural networks (RNNs) [18].

In [19], vibration one-dimensional time sequences are converted by using wavelet analysis to time-frequency images (frequency variations along time are more intimately described compared to the STFT). These images are the input of a deep convolutional neural network that extracts abstract features in order to perform fault classification (three faults: a slight crack, a medium-sized crack at the root of one tooth, and a broken tooth) with an accuracy higher than 99.5%.

A gear fault diagnosis method that combines vibration and sound signals supported by a fusion method based on improved evidence theory (IDS theory) is presented in [20]. The signals were collected in a semi-anechoic room. The vibration signal was pre-processed into complex Morlet wavelet time-frequency maps and sent to the adaptive stacked CNN. The sound signal was directly sliced and sent to the end-to-end stacked convolution neural network. The multi-sensor fusion algorithm was used to fuse the diagnosis output of vibration and sound signals, resulting in a 97.7% average fault recognition from four states (normal, worn, broken, and pitting) of the gear.

In [21], the original acceleration signal is converted into grayscale images (pixel values from 0 to 255), obtaining various textures in order to classify faults in bearings. Different fault sizes (artificial holes of different diameters performed in the inner ring using laser drilling), different fault types (inner ring fault, outer ring fault, and ball fault), and several rotational speeds are considered for the bearings. Based on the local binary pattern (LBP), texture features are extracted and used for fault classification by applying different machine learning methods (K-NN, Random Forest, NaiveBayes, BayesNet, and ANN), obtaining good performance rates from 75% to 100%.

The proposed method in [22] aims to detect gear pitting faults by training a 1D-CNN with raw acoustic emission signals and a gated recurrent unit network with raw vibration signals. The features extracted by the two networks are concatenated and used further in a deep-learning classification model. Seven different gear pitting conditions are used to test the model, obtaining a detection accuracy higher than 98%. Acoustic emission techniques are also used for 3D localization of damage in materials based on the radiation pattern of acoustic emission sources [23]. Structural crack (defect) detection methodology through thermography by generating ultrasound excitation that produces localized heating (caused by damping) into the structure is presented in [24].

In [25], a deep (multiple hidden layers) sparse autoencoder network (self-supervised machine learning) for gear pitting detection is used. The stacked autoencoder performs the sparse dictionary learning and extracts features from raw vibration data automatically. The proposed method combines the advantage of dictionary learning in sparse coding as an efficient data representation with the self-learning power of the deep sparse autoencoder for feature extraction. A single-stage gearbox reducer with spur gears is used for testing. The pitting fault (a row of circular dents of 0.5 mm depth about the pitch line of the active flank) on the driven gear is artificially introduced using an electrical discharge machine.

Gear-tooth crack fault detection is mentioned in [26]. The list of gear fault detections using deep neural networks can continue with more examples. Aspects related to the computational complexity for both the forward and back propagation processes are observed in [27], computing the total number of operations at each 1D-CNN layer (sub-sampling/down-sampling has a negligible computational cost).

Within the research context of gear defect detection, the novelty of the present paper consists of a comparative study regarding the relevance of the vibration signals on the individual axes of a triaxial accelerometer and on all three axes in the detection of four states of a gearbox with helical gears, regardless of the three different working speeds and three load levels used in the experiments. The proposed CNN-based defect detection models use 1D and 2D grayscale images representing the vibration image of data acquired on one and all three axes of the accelerometer to extract significant features capable of distinguishing between the four states under observation.

## 3. The Test Rig and the Gearbox Defects

This section is dedicated to the presentation of the test rig employed in the experiments, the vibration data acquisition system, and the description of the defects of the pinion belonging to the first stage of the reducer.

### 3.1. Test Rig Description

**Figure 2:**
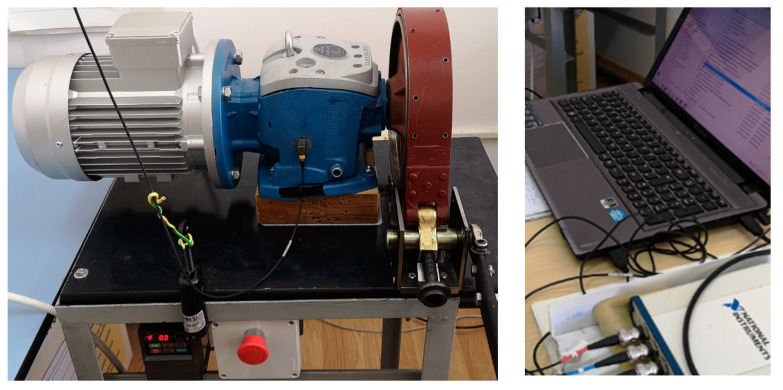
Test rig (**left**) and acquisition system (**right**).

For gear defect detection, a test rig has been designed and assembled. The experimental test rig comprised a three-phase AC motor, a variable frequency drive (VFD) as a motor speed regulator, a speed reducer gearbox, and a dry friction brake (Figure 2, left).

**Figure 3:**
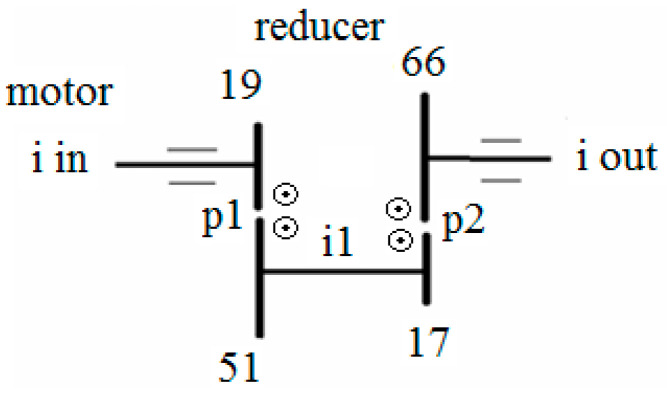
Speed reducer kinematic chain and the number of teeth for each gear wheel.

The two stages of the speed reducer with two gear pairs, p1 (P1, G1) and p2, and the parallel shafts are schematized in Figure 3, where each of the four arrowheads shows the direction of rotation of the particular gear wheel by indicating the side of the gear (relative to its rotation axis) which is coming toward the reader.

The actual speed reducer ratio *ir* = *i_in_*/*i_out_* is 10.421. The relation between the input and output turning speeds is expressed in Equation (1).

(1) $$i_{in}\times \frac{19}{51}\times \frac{17}{66}=i_{out}\;\mathrm{or}\;\frac{i_{in}}{10.421}=i_{out}$$

**Figure 4:**
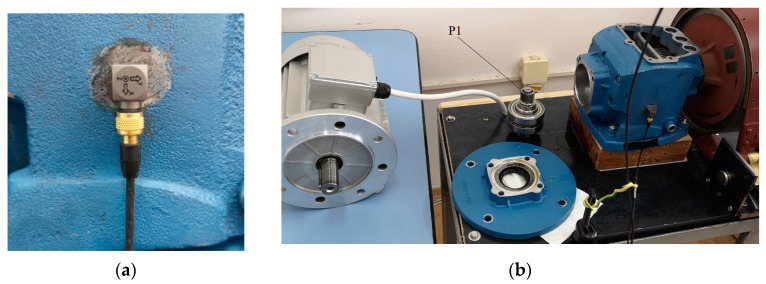
(**a**) Triaxial accelerometer; (**b**) input shaft with pinion P1 and two bearings, input flange, and reducer top cover removed.

A triaxial B&K piezoelectric accelerometer with integral electronics was glued on the reducer cast iron housing lateral surface. The sensor sensitivity is about 10 mV/g, with small variations on each accelerometer axis. The attachment surface was previously prepared, removing paint and made it flat by polishing (Figure 4). The X-axis of the accelerometer is perpendicular to the base of the reducer (normal to the input shaft), pointing downwards; the Y-axis is along the reducer input shaft and pointing to the right, and the Z-axis is perpendicular to the lateral reducer gluing surface. The National Instruments USB-4431 five-channel dynamic signal acquisition board, USB connected to a laptop (Figure 2, right), and a LabVIEW application are employed for the acquisition from the accelerometer using the first three analog input channels. The fourth input channel is used for the tachometer input. All four input channels of the acquisition board are simultaneously sampled, with 24-bit resolution and support sample rates of up to 204.8 kS/s.

The selected nominal rotating speeds of the AC motor were 1350 rpm, 1375 rpm, and 1400 rpm for each of the four states of the reducer: the healthy state, pitting on a pinion tooth, localized wear on a tooth flank, and low level of oil. The friction brake is used to impose three load levels: the unloaded state (L1) and two different friction levels (L2, L3), using fixed lever positions associated with the friction brake. The three load levels are applied for each rotating speed and each of the four states.

### 3.2. Defects on the Helical Pinion

In a helical gear, more than one tooth pair is in contact during mesh, being able to carry a larger load than the spur gears. Each pair of meshing teeth has a contact line on the active flank pair. The sum of the contact line lengths is important to establish the capable thrust load of the gear pair; an increased length diminishes the effect of a local pitting defect. The sum of the contact line lengths also varies slightly during the gear meshing cycle. Therefore, the helical gears operate smoother and quieter than spur gears. The sliding velocity between active flanks is the relative tangential velocity of the tooth profiles. This velocity is relatively low at a pitting defect located close to the pitch diameter. At the pitch point, the relative velocity is zero and increases as the distance from the pitch point increases in the line of action. Being small in the middle of the tooth, the sliding velocity cannot lead to scuffing defects. The helix angle β, between the pinion axis of rotation and tooth orientation, on the observed pinion (P1) is relatively large (for spur gear β = 0). Two of the defects (pitting and localized wear) belong to the pinion P1 of the p1 gear pair. The pinion P1 and the two bearings mounted on the reducer input shaft extracted from the housing are shown in Figure 4.

The present study considers four health states of gear condition, denoted by D1, D2, D3, and D4, as depicted in Table 1. D2 is the normal, healthy state with no defect. D1 represents an unhealthy state, with poor lubrication, in which the oil level is low in the reducer. Only the teeth of the wheel (positioned under the pinion) are immersed in lubricant. The structural defects corresponding to the D3 and D4 states are described in the following sections.

#### 3.2.1. Gear Pitting Defect

Pitting is the most common failure mode for gear teeth and takes macro or microforms. Macro-pitting refers to pits larger than 1 mm in diameter. Pitting usually occurs in a narrow band at the pitch line or slightly below the pitch line.

Contact fatigue of steel gears tooth flanks generates pitting by removing material from the tooth surface. The process is influenced by the lubrication (oil viscosity and temperature), working loads, specific sliding between contact flanks, flank surface roughness and shape, and gear material in terms of the S-N curve (alternating stress level and the number of cycles to failure). The hydraulic pressure of the oil during the mesh acts in the crack and causes the cracks to grow into pits. Pitting becomes a local defect and of small dimension. Because pitting leads to higher pressure and causes stress concentration on the unpitted surface, pits will finally spread to neighboring regions, covering the entire dedendum of the tooth flanks. Pitting begins near the pitch point where a high friction force is present due to the low sliding velocity. Pitting is usually shell-shaped and localized predominantly in the areas of negative specific sliding.

**Figure 5:**
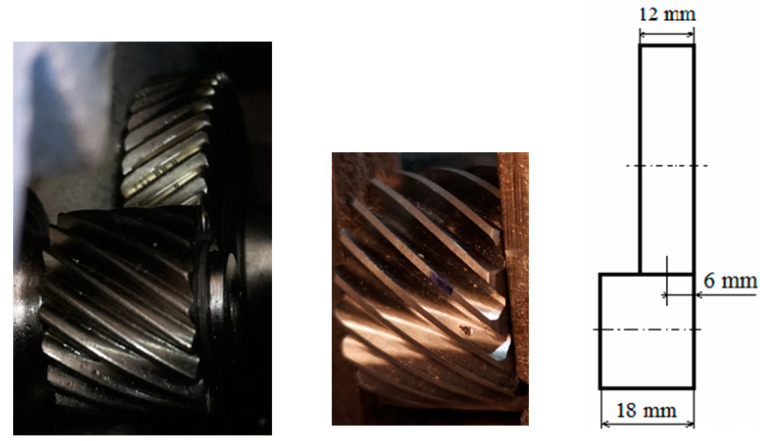
p1 (P1, G1) gear pair (**left**), pitting defect on pinion P1 (**middle**) and defect position (**right**).

Pinion P1 (19 teeth) with the pitting defect meshes with gear G1 (51 teeth). The defect is located approximately 6 mm away from the right side (Figure 5) and placed at mid-width of G1. In Figure 3, the direction of rotation of the gears during the test can be observed. Corroborating with Figure 5, middle, one can observe that the artificial pitting defect is placed on the active/contact flank of the tooth.

#### 3.2.2. Localized Wear Tooth Defect

**Figure 6:**
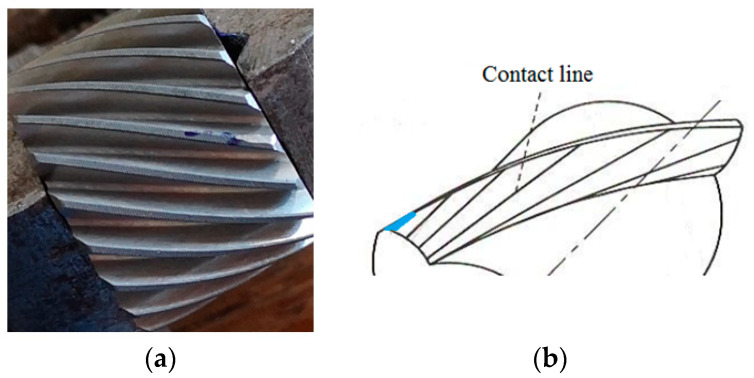
Pinion P1: (**a**) localized wear; (**b**) successive contact lines and the wear location in blue.

Wear on the teeth flanks is the material loss from the engaging surfaces due to the sliding and rolling motions. When two of the teeth start to engage, the contact is gradual, starting at one end of the tooth and maintaining contact as the gear rotates into full engagement. The considered defect is a transverse profile slope modification at the addendum of the unhealthy tooth of the helical pinion P1 (19 teeth). The effect of tooth wear (the chamfer) is the shortening of the contact line when the pinion faulty tooth area has meshed with the G1 wheel. The wear is 3 mm long on the helical tooth out of the total 18 mm width of the pinion (Figure 6). This fault changes the gear dynamics, and the associated vibration is an efficient real-time assessment of the contact in gear. The helical gear transmission ratio is less sensitive to wear fault in comparison to spur gear because of the increased length of the contact lines associated with the number of pairs of teeth found in contact (contact ratios) for helical gear. It has been noted that the higher-order harmonics are more sensitive to tooth wear in comparison to the tooth meshing frequency.

## 4. Data Acquisition and Data Processing

**Figure 7:**
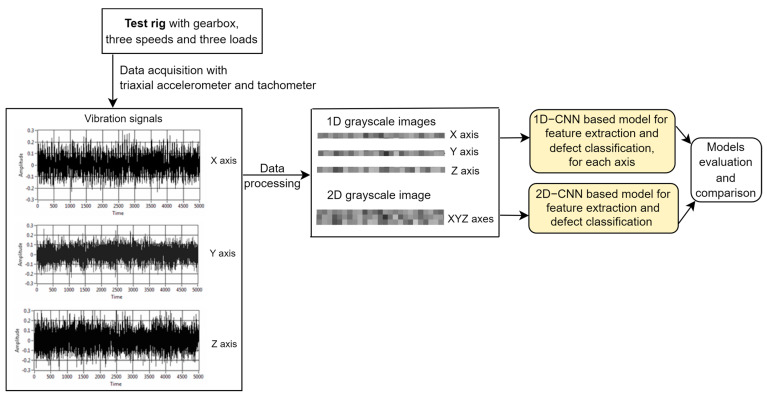
Methodology.

In this section, the first two steps of the methodology (depicted in Figure 7) proposed to achieve the research goal RG1 are described. The data acquisition is performed using a LabVIEW application.

The aim of the data processing step is to obtain from the raw vibration signals 1D and 2D grayscale images used further to build CNN-based defect detection models. These deep learning models will learn from the images significant features that distinguish the four gearbox states (defects), and then they are used in defect detection (classification). The defect detection task is modeled as a multi-class classification problem, the classes corresponding to the four system states (D1, D2, D3, and D4).

### 4.1. Data Acquisition

A LabVIEW application was created for the acquisition of the three axes accelerometer signals and the associated tachometer signals, which were saved in 36 files. For each health state, three rotation speeds were selected for each load level out of the three different loads, resulting in 9 files per health state. From these files, signals for the accelerometer axes (X, Y, and Z) are extracted and processed individually or grouped using MATLAB.

The rotation speed of the reducer output *i_out_* is measured by the tachometer, and considering the reducer ratio in Equation (1), the pinion P1 rotational speed *i_in_* is derived. The following three reducer output rotational speeds, 2.16 rps, 2.20 rps, and 2.24 rps, were employed, situated at the higher limit of the motor capability. The pinion and the associated AC motor speeds are presented in Table 2.

The sampling frequency of the acquisition is F_s_ = 10 kHz, and the duration of one record is 240 s, resulting in 2,400,000 samples per axis for one of the three rotational speeds and one of the three specific loads.

### 4.2. Data Processing

For each axis (X, Y, and Z), a number of about 300 comma separated values (csv) files were generated, resulting in 36 folders associated with 4 states × 3 loads × 3 velocities. The files pertaining to the same state were collected together, obtaining about 2700 files per fault for one accelerometer axis. The csv files were converted into png files in order to store data and feed the CNNs. The acquired time domain vibration (acceleration) signal is converted into a grayscale image of 16-bit depth (the brightness range of 65,536 levels). The vibration signal was previously rectified; hence, the negative values became positive.

Considering the constant acquisition sampling frequency (F_s_ = 10 kHz), for a lower input shaft rotation speed (longer time per revolution), a larger number of samples are acquired for a P1 pinion complete rotation, as presented in Table 3.

We proposed an integer number of pinion P1 rotations in a line of a png file. The best classification results were obtained for a larger number of pinion rotations, namely for 18 rotations, regardless of the motor velocity (18 rotations of the pinion P1 means about 1.8 rotations at the reducer output shaft). The total number of samples of a record is divided by the number of samples in 18 rotations. For v1, v2, and v3 shaft velocities, 300, 305, and 311, png files are generated. The MATLAB function *mat2gray* is used to convert a numerical matrix containing the acquired samples to a grayscale image (brightness range of the image) that contains values in the range of 0 (black) to 1 (white).

For the first approach, we propose 1D grayscale images to feed the CNN and to build a 1D-CNN-based model. A png file contains samples from one accelerometer axis (X, Y, or Z) on a single line. The samples came from 18 rotations of P1, rounding to the closest multiple of 3 as follows: 7996.7 samples rounded to 7995 samples for velocity v1, 7851.3 samples rounded to 7851 samples for velocity v2, and 7711.1 samples rounded to 7710 samples for velocity v3. The files (png) for the v1 (lowest) and v2 (medium) rotation speeds, with 7995 or 7851 samples per line, were scaled using the *imresize* MATLAB function to have the same size, 7710 samples, for the png files for all velocities (Table 4). The png files were collected together for the three velocities and three loads, with the same size and belonging to the same system state (defect).

Three data sets, Sx, Sy, and Sz, corresponding to the vibration signals measured on individual axes, X, Y, and Z, were generated and used further as input data in the 1D-CNN-based defect detection models. Each set contained a total of 9909 png files, 1D grayscale images, from which 2592 images were for the D1 state and 2439 for each of the other states: D2, D3, and D4.

In the second approach, we considered a 2D grayscale image with samples from all three axes (X, Y, and Z) of the accelerometer in one file. The first line of the file contains samples from the X-axis collected from 18 rotations of the P1 pinion. Similarly, the second and the third lines contain samples from 18 rotations of P1 collected from the Y-axis and Z-axis, respectively. The number of samples in one png file for the higher P1 rotational speed (v3) is shown in Table 5.

**Figure 8:**
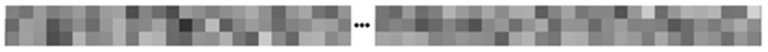
2D grayscale image containing data from all three accelerometer axes.

An example of a png file with three lines is shown in Figure 8. The number of png files per state varies slightly because a couple of records are shorter in time, namely for the ones with the L3 load.

The generated 2D images [3, 7710], corresponding to the accelerometer data measured on all three axes, X, Y, and Z, will form the Sxyz data set. This set contains 2592 images for the D1 state (class) and 2439 for each of the other states (classes): D2, D3, and D4, so a total of 9909 png files.

## 5. Proposed CNN-Based Defect Detection Models

In this section, theoretical aspects of convolutional neural networks (CNNs) are introduced, and then the proposed 1D-CNN and 2D-CNN-based classification models designed to detect the four states (classes) of the gearbox are described. The vibration information measured on only one of the three axes (X, Y, and Z) and transformed into 1D grayscale images are used to build three 1D-CNN-based models: Mx, My, and Mz. The information extracted by the triaxial accelerometer on all three axes, represented as 2D grayscale images, is the input of Mxyz, a 2D-CNN-based model.

### 5.1. CNN Model

A convolutional neural network (CNN) [28] is a deep artificial neural network that uses convolution (a mathematical operation: the dot product of two matrices) in some of its hidden layers. The aim of the CNN is to extract step by step the important features from input data that has a grid-like topology, such as time-series data (1D grid containing samples at regular time intervals) and image data (2D grid of pixels or 1D grid of pixels).

The architecture of a classification system based on CNN consists of two stages: (1) *Feature extraction*—from an input image, a feature vector is extracted, and it is used further in (2) *Classification*. To learn the significant features of the input images one or more blocks of the following layers (*convolutional-normalization-activation-pooling*) are included in the system architecture.

- *Convolutional layer*. To extract different properties/features of the input image, more kernels/filters (small matrices initialized with random values and then learned during the training process) are used in convolution operations with the aim of obtaining feature maps. The filters define the receptive field of the neurons. A filter (*F*, of size *M* × *N*) slides left-to-right and top-down over the initial matrix (*Im*, image) with a predefined stride (*s*), and a convolution (dot product) is applied to the filter and the selected region (patch) of the input matrix, resulting a matrix called feature map (*Fm*). If a corresponding padding operation is applied to the initial matrix (*Im*), and *s =* 1, then *Fm* has the same size as *Im.* The elements of *Fm* are calculated using Equation (2). The hyperparameters of this layer are the number of filters, the kernel/filter size, the stride size, and the padding type.
  (2) $$Fm(i,j)=(F*Im)(i,j)=\sum_{m=0}^{M-1}\sum_{n=0}^{N-1}Im(s\cdot i+m,s\cdot j+n)\;F(m,n)$$
- *Batch normalization layer*: the elements of the previous layer are normalized (re-centered and re-scaled) based on the statistics within each mini-batch before the application of the activation function.
- *Activation layer*: a non-linear function is applied to the neuron’s input. We chose to use in our models the rectified linear units (ReLU) because this activation function is non-saturated and speeds up the convergence of the gradient descent towards the global minimum of the loss function. ReLU(x) = *max*{0,x}. A neuron is activated only if the output of the transformation is greater than 0, resulting in greater computational efficiency than using other activation functions such as *sigmoid* and *tanh*.
- *Pooling layer*. The feature maps are reduced (down-sampling operation) by applying max or average operations to a region obtained by sliding a filter (window) with a stride over the input matrix. The hyperparameters of this layer correspond to the pooling filter size and the pooling stride size.

The output feature maps of the last block (*convolutional-normalization-activation-pooling*) are concatenated over the filters, and a flatten layer is generated, representing the feature vector learned during the described process.

In the classification stage, the input is the feature vector that is further reduced step by step using intermediate fully connected layers, the last one, *z* = (*z*_1_, …, *z_K_*), corresponding to the number (*K*) of classes. This layer is followed by a *softmax layer* containing the probability distribution over the set of predefined classes. These probabilities are calculated using the softmax function (normalized exponential function) from Equation (3)

(3) $$p_{i}=\sigma {\left(z\right)}_{i}=\frac{e^{z_{i}}}{\sum_{j=1}^{K}e^{z_{j}}},\;\mathrm{where}\;i=1,\ldots ,K\;\mathrm{and}\;z=(z_{1},\ldots ,z_{K})\in R^{K}$$

A training stage is needed to build a CNN-based model using a large amount of input data and the corresponding output values. The aim of the training process is to improve the model accuracy so as to minimize a loss function by iteratively adjusting the model parameters (the weights and the filter values). By comparing the actual and the predicted output values, a loss function measures the model fitting.

In classification problems, the cross-entropy (logarithmic) loss function decreases as the predicted class probability converges to the actual class output (0 or 1). For *K* output classes, (*p*_1_, …, *p_K_*) the softmax predicted probabilities and (*c*_1_, …, *c_K_*) the one-hot encoding of the actual classes output, the formula for the cross-entropy loss is defined in Equation (4).

(4) $$L_{CE}=-\sum_{i=1}^{K}c_{i}\log(p_{i})$$

### 5.2. Model Based on 1D-CNN for Data from One Accelerometer Axis

To achieve the research goal RG2, a 1D-CNN-based classification model is introduced to analyze accelerometer data separately for each axis. From a 1D grayscale image [1, 7710], relevant and discriminative features are extracted and used further in the detection of the four states (classes).

**Figure 9:**
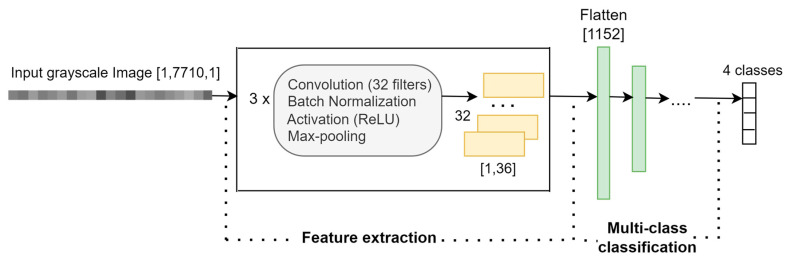
Architecture of the 1D-CNN-based classification model.

Building three classification models, Mx, My, and Mz, based on the same architecture and using vibration information measured on the corresponding axes, X, Y, and Z, we compare their efficiency in discriminating between the four states. The architecture of the 1D-CNN proposed model is depicted in Figure 9.

In the *feature extraction* phase, three blocks of *convolutional-normalization-activation-pooling* layers are used to extract features from the 1D grayscale image, and 32 feature maps of size [1, 36] are generated. The flatten layer represents the feature vector and is obtained by concatenating the feature maps. In the *classification phase*, the feature vector is reduced, using three fully connected layers, to a vector of 4 elements corresponding to the four classes (system’s health states). The architecture of the 1D-CNN-based model is implemented in MATLAB R2023a using the functions that create different layer types.

Table 6 contains a detailed description of all the layers of the 1D-CNN-based model and the corresponding hyperparameters. The convolution and pooling filters slide along one dimension, corresponding to time.

### 5.3. Model Based on 2D-CNN for Data from All Three Accelerometer Axes

**Figure 10:**
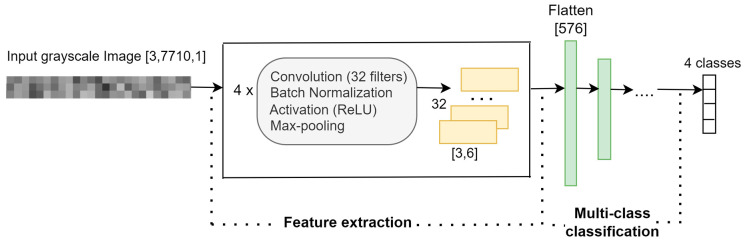
The architecture of the 2D-CNN-based classification model.

For the research goal RG3, the proposed architecture of the 2D-CNN-based classification model, Mxyz, is depicted in Figure 10. The input is a 2D grayscale image of size [3, 7710], where the three lines correspond to the signals measured simultaneously on the three axes, X, Y, and Z.

Using four blocks of *convolutional-normalization-activation-pooling* layers, 32 feature maps of size [3, 6] are extracted from the 2D grayscale image. These maps are concatenated into a feature vector of 576 elements, the input in the classification phase. The 4-class classification is performed using two fully connected layers of 128 and 4 neurons, followed by a softmax layer and the class output layer. The architecture of the 2D-CNN-based model is implemented in MATLAB R2023a using the functions that create different layer types.

The description of all the layers of the model and the corresponding hyperparameters are presented in Table 7. In the proposed model, the pooling filters slide along the time dimension.

## 6. Experiments and Discussion

This section provides the results of the performed experiments in MATLAB R2023a, using all four classification models (Mx, My, Mz, and Mxyz), and conclusions related to the research goals, RG2 and RG3, are drawn. The data sets Sx, Sy, and Sz corresponding to the individual vibration signals on the X, Y, Z axes, and Sxyz, containing the signals on all three axes, are used in the experiments. Each set contains a total of 9909 grayscale images, from which 2592 images are for D1, and 2439 are for each of the other three states: D2, D3, and D4. The 1D images from the Sx, Sy, and Sz data sets correspond to the accelerometer data acquired from individual axes, X, Y, and Z, respectively. The 2D images from Sxyz represent the accelerometer data measured simultaneously on all three axes. The CNN-based classification models, Mx, My, Mz, and Mxyz, have been built and tested using the corresponding data sets in a proportion of 85% and 15%, respectively. Therefore, 8422 instances (85%) were used to build the models, which were then tested for 1487 instances (15%).

The implementation options for building all four models are as follows:

- 8422 images, from which 90% were for training and 10% for validation; shuffle every epoch;
- Stochastic gradient descent with momentum (sgdm) optimizer;
- Maximum number of epochs: 60;
- Mini-batch size: 126 instances, 60 iterations per epoch;
- Validation frequency: 60 iterations;
- Learning rate: 0.01;
- Output/final model chosen based on the best validation loss criterion.

According to the models’ implementation options presented above, the training and validation phases performed in our experiments can be described as follows.

1. The training and validation are performed in 60 epochs.
2. In each epoch, the data set used to build the model is randomly split into a training data set (90%) and a validation data set (10%).
  - The training set is split into mini-batches of 126 instances (input images, actual output class), and 60 iterations per epoch are performed.
  - In each iteration, 126 images (a mini-batch) are used, one at a time, to feed the current CNN-based model and to obtain the predicted output class for each input. The training loss for the current iteration is calculated from the losses obtained for all instances used in the iteration.
  - The weights and the filters’ values are updated based on the training loss of the current iteration, and a new model is generated and used in the next iteration.
  - After 60 iterations are performed, at the end of the epoch, the performance of the last model is evaluated using the validation data set of that epoch. The validation loss and validation accuracy are calculated.
3. The learning process during the 60 epochs (3600 iterations) is observed on the loss and accuracy curves obtained using the corresponding values for training (at each iteration) and for validation (at the end of the epoch). Such curves are presented in the next sections. We can choose to perform validation more frequently during an epoch, for example, after every 30 iterations, to have more values for validation loss and validation accuracy in the curves.
4. After 60 epochs are performed, the model with the best validation loss is chosen as the final/output model, which is further used in testing.

The performance of the proposed models is evaluated on the testing data set, and it is measured by the testing accuracy metric, which is the percentage of the instances correctly classified out of all the tested instances.

### 6.1. Comparison between the 1D-CNN-Based Models

For the proposed comparison in RG2, the experiments were conducted on the Sx, Sy, and Sz sets, aiming to identify which of the three data sets provides the most relevant information to correctly detect the classes D1, D2, D3, and D4.

The learning curves for the accuracy metric, corresponding to one run of the Mx, My, and Mz models, are depicted in the figures from Table 8. We conclude that the features for discriminating the four classes are learned faster in the training process from the information extracted on the Y axis. Also, the vibration signal measured on the Z axis is less informative compared with the signals from the other two accelerometer axes, and more learning time is needed to extract significant features that distinguish the speed reducer states under observation.

To evaluate the 1D-CNN-based models (Mx, My, and Mz) we performed 10 runs for each model. In a run, the subsets (training/validation/testing) from Sx, Sy, and Sz were selected randomly according to the percentages previously presented. The analysis of the learning and loss curves showed that the validation accuracy of 95% is reached in 27–33 epochs by Mx, in 10–13 epochs by My, and in 27–33 epochs by Mz. After reaching 95% accuracy, there is a plateau with small variations, the final model being generated after 40–50 epochs.

Table 9 presents the learning and testing metrics for the classification models, Mx, My, and Mz, expressed as confidence intervals, $CI=ma\pm 1.96\;\sigma /\sqrt{n}$ [29], at the 95% confidence level over *n* = 10 runs, where *ma* is the mean value and σ is the standard deviation. These testing results are in agreement with the results obtained in the learning process (training + validation); the testing accuracies are very close to the validation accuracies, and the validation losses are very small.

All three models achieved very good results; the testing accuracies were higher or equal to 97%, and the best-performing model was My, with a mean testing accuracy of 98.91%. The conclusion related to RG2 is that the most relevant information that discriminates between the four classes (states) is provided by the vibration data measured on the Y accelerometer axis along the reducer input shaft direction.

The accuracy is an overall performance metric, but the precision, recall, and F1-score measures are used to evaluate a model’s performance for each class. Precision and recall are inversely related, so a good classification model needs to strike the right balance between these two measures. The F1-score is defined as the harmonic mean of precision and recall, and a performing model should achieve an F1-score close to 1.

We continue the comparison of the performances of the three models (Mx, My, and Mz) at the level of classes. For each model, 10 runs were performed, and a global confusion matrix was calculated by summing up the 10 confusion matrices generated. Based on this matrix, the precision, recall, and F1-score were computed. Table 10 contains the three performance metrics per class for all three models in 10 runs.

By analyzing the three global confusion matrices corresponding to the three models, we observed that from the perspective of confusion at prediction, in almost all the cases of misclassification, there was confusion between D1 and D2 classes, and between D3 and D4 classes. The results in Table 10 show a right balance between precision and recall values for all classes in all three models.

In the model Mx, with 97.15% accuracy, the F1-score is around 97% for all four classes, and D1 and D2 are slightly better detectable than D3 and D4. In the best classification model, My, with 98.91% accuracy, the F1-score is around 99% for three classes, and D2 is the least detectable class (F1-score = 98.58%).

In the least performing model, Mz, with 97% accuracy, the classes D3 and D4 are better detectable than D1 and D2. The F1-scores of D3 and D4 are higher by about 3% than the scores of the other two classes. We also remark that the F1-scores for D3 and D4 in Mz are higher by 1.63% and 1.44%, respectively, than the corresponding scores in Mx.

We conclude that in the models Mx and My, the vibration signal measured on the X and Y axes does not favor the detection of a defect (state) over the others. The vibration signal on the Z axis contains information that better detects structural defects (D3, D4) than the other two states (D1, D2). In the case of measurement with a uniaxial accelerometer, it will be oriented along the Y axis.

### 6.2. Evaluation of 2D-CNN-Based Model

The 2D-CNN-based model, Mxyz, was evaluated by performing 10 runs. In each run, the Sxyz dataset was randomly split based on the following percentages: for building the model, 85% (from which 90% for training and 10% for validation), and for testing, 15%. The values of the learning and the testing metrics of the Mxyz model expressed as confidence intervals (*CI*) at the 95% confidence level, are presented in Table 11. The mean testing accuracy achieved by Mxyz is 99.63%, exceeding by 0.72% the performance of My, the best-performing 1D-CNN-based model.

From the perspective of the learning process, after analyzing the learning curves for accuracy in all 10 runs, the conclusion was that the validation accuracy of 95% is reached in 4–6 epochs and 99% accuracy after 20 epochs. Working with all three signals measured by the triaxial accelerometer helps the model learn the relevant features that discriminate between the four defects much faster and more accurately than working with the signal extracted from only one axis.

We present in Table 12 the learning process of Mxyz in one run, represented by the learning curve of accuracy and the loss curve. These curves indicate a good fit model; in both pairs ((training loss, validation loss) and (training accuracy, validation accuracy)), the metrics values are close to each other. We remark on a steep portion of the learning curve in the first four epochs, corresponding to an initial period of fast learning, the model achieving around 95% validation accuracy. In the next epochs, the learning process continues with a moderate improvement in performance until 99% validation accuracy is reached in 20 epochs, and then there is a plateau for accuracy and loss, with small variations for the rest of the epochs. Based on the best validation loss criterion, the final model is obtained in the 33rd epoch.

The testing results obtained using the previously described Mxyz model are shown in Table 13 using the confusion matrix. Achieving a 99.53% testing accuracy from 1487 unseen instances, only 7 of them have been misclassified by the model. All the instances of the D1 class were correctly identified, one instance of D2 was misclassified as D1, and there was confusion in the detection of 6 instances of D3 and D4 classes, corresponding to the structural defects of the gearbox.

The CNN-based detection models proposed in this paper proved to perform very well in the detection of the gear states (low oil level, normal, pitting, wear), achieving 97%, 97.15%, 98.91%, and 99.63% mean testing accuracies, equaling or outperforming models that identify similar gear defects. For each case, the performance is influenced by particular aspects regarding the geometry and positioning of the defect on the flanks, gear type, working conditions, and the overall dynamics of the investigated mechanical system. Compared with other approaches, in our study, we analyzed the individual vibration signals on the X, Y, and Z axes, measured simultaneously by the accelerometer, with the goal of identifying which axis (relative to the reducer input shaft with a helical pinion) provides the most relevant information capable of distinguishing the four states under observation. After we studied the individual signals on the three axes, we employed the full potential of the triaxial accelerometer to increase accuracy in detecting gear states. Another difference in the present approach from other studies is that the four states are successfully detected regardless of the three different working speeds and three load levels used in the experiments.

## 7. Conclusions and Future Work

A testing rig for gear fault diagnosis was built comprising a speed reducer gearbox with helical gears, a three-phase AC motor, a motor speed regulator, and a friction brake. Aspects about the onset mechanism of some gear defects are discussed, namely pitting and localized wear on a helical pinion. Raw vibration signals measured by a triaxial accelerometer for three different working speeds and three load levels are used to detect one of the four condition states of the gearbox under observation. These four states correspond to a low lubricant level on the speed reducer, the normal state, and two structural defects: localized small pitting on a helical pinion tooth flank and localized wear on the helical pinion. For defect detection, deep-learning CNN-based models were proposed to extract relevant vibration features capable of distinguishing between the four states of the gearbox.

The present paper is a study on the mentioned gearbox defect detection models based on three proposed research goals.

The first research goal was achieved by proposing and implementing a methodology that solves the defect detection problem for a speed reducer with helical gears (four health states), regardless of the selected load level or speed level on the test rig, using deep learning CNN-based models built from raw vibration signals.

Three 1D-CNN-based models (with the same architecture) were trained on the vibration signal from each accelerometer axis, aiming to observe the efficiency of each axis to the defect detection problem, as proposed in the second research goal. The best-performing model, with a testing accuracy of 98.91%, is based on the signal measured on the Y axis along the reducer input shaft direction. The information provided by the vibration data from the X and Z axes is less relevant in discriminating the four classes, with the corresponding models achieving a mean accuracy of 97.15% and 97%, respectively. The model My, is recommended when a uniaxial accelerometer is employed.

A 2D-CNN-based model, built using the data acquired from all three axes of the accelerometer, was employed to extract significant vibration features that are used to identify the state of the gearbox (the third research goal). The mean testing accuracy achieved by this model is 99.63%, exceeding by 0.72% the performance of the best-performing 1D-CNN-based model for the Y axis.

The performances of the proposed models equal or outperform the performances of other models in the literature that detect similar gear defects. The future work will observe gear defects in both reducer helical gear stages for various loads and velocity combinations.

## Figures and Tables

**Table 1:** List of helical gearbox defects (health states).

State (Defect)	Description of the Helical Gearbox Defect (State)
D1	Low oil level
D2	Normal, no defect
D3	Pitting on a pinion tooth flank
D4	Localized wear on a pinion tooth flank

**Table 2:** Pinion P1 and AC motor rotation speeds.

Nominal Speed Values	v1	v2	v3
Reducer taho velocity: *i_out_* [rpm]	129.6	132	134.4
Reducer velocity: *i_out_* [rps]	2.16	2.20	2.24
P1 (AC motor) speed: *i_in_* [rps]	22.51	22.93	23.34
P1 (AC motor) speed: *i_in_* [rpm]	1350.4	1375.4	1400.4

**Table 3:** Number of samples per one pinion (P1) rotation.

Samples per one rotation = Sampling frequency (F_s_)/P1 rotation speed [rps]			
P1 rotation speed [rps]:	22.51 (v1)	22.93 (v2)	23.34 (v3)
Samples per one P1 rotation:	444.26	436.18	428.39

**Table 4:** Image generation for data from only one accelerometer axis (X, Y, or Z).

1D grayscale image [1, 7710]	7710 samples from 18 rot. of P1, X axis	one png file
1D grayscale image [1, 7710]	7710 samples from 18 rot. of P1, Y axis	one png file
1D grayscale image [1, 7710]	7710 samples from 18 rot. of P1, Z axis	one png file

**Table 5:** Image generation for data from all three accelerometer axes.

Line 1	7710 samples from 18 rot. X axis	one png file containing 18 rot. of P1 2D grayscale image: [3, 7710]
Line 2	7710 samples from 18 rot. Y axis	
Line 3	7710 samples from 18 rot. Z axis	

**Table 6:** Description of the 1D-CNN-based model.

	**Input Layer:**	1D grayscale image			[1, 7710, 1]
**Feature extraction:** three blocks of *convolutional-normalization-activation-pooling* layers					
	**Layers**	**Hyperparameters**			**Feature maps**
Block 1	Convolutional	filters: 32	filter size: [1, 9]	stride size: [1, 1]	32 × [1, 7710]
	Batch normalization				32 × [1, 7710]
	Activation	ReLU			32 × [1, 7710]
	Pooling	max	filter size: [1, 6]	stride size: [1, 6]	32 × [1, 1285]
Block 2	Convolution	filters: 32	filter size: [1, 9]	stride size: [1, 1]	32 × [1, 1285]
	Batch normalization				32 × [1, 1285]
	Activation	ReLU			32 × [1, 1285]
	Pooling	max	filter size: [1, 6]	stride size: [1, 6]	32 × [1, 215]
Block 3	Convolutional	filters: 32	filter size: [1, 9]	stride size: [1, 1]	32 × [1, 215]
	Batch normalization				32 × [1, 215]
	Activation	ReLU			32 × [1, 215]
	Pooling	max	filter size: [1, 6]	stride size: [1, 6]	32 × [1, 36]
	Flatten layer				[1152]
**4—class** **classification**	Fully connected layer				[256]
	Fully connected layer				[128]
	Fully connected layer				[4]
	Softmax layer				[4]
	Classoutput layer	Cross-entropy loss function			[4]

**Table 7:** Description of the 2D-CNN-based model.

	**Input Layer:**	2D grayscale image			[3, 7710, 1]
**Feature extraction:** four blocks of *convolutional-normalization-activation-pooling* layers					
	**Layers**	**Hyperparameters**			**Feature maps**
Block 1	Convolution	filters: 32	filter size: [3, 3]	stride size: [1, 1]	32 × [3, 7710]
	Batch normalization				32 × [3, 7710]
	Activation	ReLU			32 × [3, 7710]
	Pooling	max	filter size: [1, 6]	stride size: [1, 6]	32 × [3, 1285]
Block 2	Convolution	filters: 32	filter size: [3, 3]	stride size: [1, 1]	32 × [3, 1285]
	Batch normalization				32 × [3, 1285]
	Activation	ReLU			32 × [3, 1285]
	Pooling	max	filter size: [1, 6]	stride size: [1, 6]	32 × [3, 215]
Block 3	Convolution	filters: 32	filter size: [3, 3]	stride size: [1, 1]	32 × [3, 215]
	Batch normalization				32 × [3, 215]
	Activation	ReLU			32 × [3, 215]
	Pooling	max	filter size: [1, 6]	stride size: [1, 6]	32 × [3, 36]
Block 4	Convolution	filters: 32	filter size: [3, 3]	stride size: [1, 1]	32 × [3, 36]
	Batch normalization				32 × [3, 36]
	Activation layer	ReLU			32 × [3, 36]
	Pooling layer	max	filter size: [1, 6]	stride size: [1, 6]	32 × [3, 6]
	Flatten layer				[576]
**4—class** **classification**	Fully connected layer				[128]
	Fully connected layer				[4]
	Softmax layer				[4]
	Classoutput layer	cross-entropy loss function			[4]

**Table 8:** Learning curves for accuracy in training and validation.

Model	Learning Curves 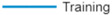 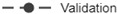
Mx	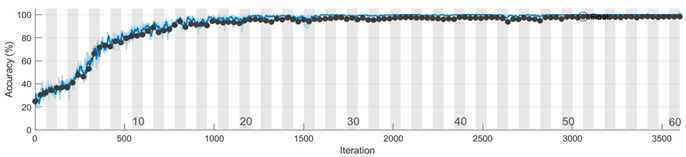
My	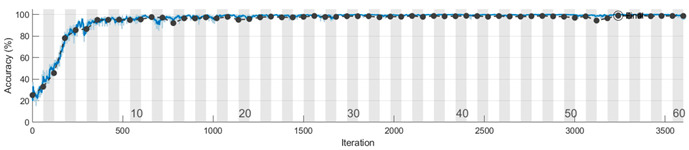
Mz	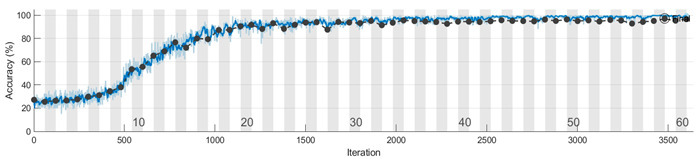

**Table 9:** 1D-CNN-based models evaluation.

Metrics	Models		
	Mx	My	Mz
Testing accuracy (*CI* %)	97.15 ± 0.38	98.91 ± 0.28	97.00 ± 0.18
Validation accuracy (*CI* %)	97.40 ± 0.59	99.00 ± 0.21	97.35 ± 0.466
Validation loss (*CI*)	0.076 ± 0.015	0.09 ± 0.083	0.082 ± 0.012
Training loss (*CI*)	0.0021 ± 0.001	0.0033 ± 0.002	0.015 ± 0.007

**Table 10:** Precision (%), recall (%), and F1 score (%) per class in all three 1D-CNN-based models.

Class	Models								
	Mx			My			Mz		
	Precision	Recall	F1-score	Precision	Recall	F1-score	Precision	Recall	F1-score
D1	97.42	97.12	97.27	99.15	98.79	98.97	95.68	95.34	95.51
D2	97.09	97.60	97.34	98.74	98.41	98.58	95.55	96.14	95.85
D3	97.22	96.53	96.87	98.73	99.48	99.10	98.10	98.91	98.50
D4	96.68	97.19	96.93	99.10	99.04	99.07	98.90	97.85	98.37

**Table 11:** Performance metrics for the 2D-CNN-based model.

**Model** **Mxyz**	**Testing Accuracy** **(*CI* %)**	**Validation Accuracy** **(*CI* %)**	**Validation Loss** **(*CI*)**	**Training Loss** **(*CI*)**
	99.63 ± 0.097	99.64 ± 0.155	0.009 ± 0.003	0.0017

**Table 12:** Learning and loss curves of the 2D-CNN-based model.

**Learning curve** 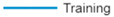 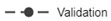
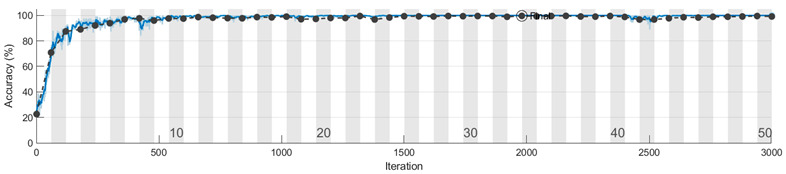
**Loss curve** 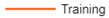 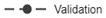
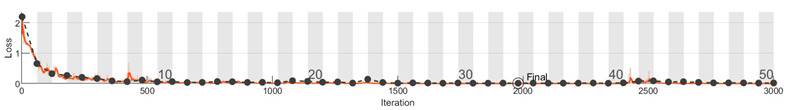

**Table 13:** Confusion matrix obtained in one run of Mxyz.

Model: Mxyz, Testing Accuracy = 99.53%
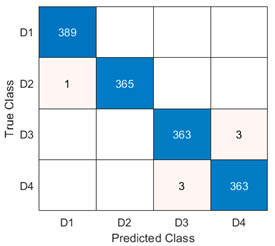

## Data Availability

The experimental vibration data are available under request.

## References

[1] Randall R.B. (2021). Vibration-Based Condition Monitoring: Industrial, Automotive and Aerospace Applications.

[2] Goldman S. (1999). Vibration Spectrum Analysis: A Practical Approach.

[3] Huangfu Y., Chen K., Ma H., Li X., Han H., Zhao Z. (2020). Meshing and dynamic characteristics analysis of spalled gear systems: A theoretical and experimental study. Mech. Syst. Signal Process..

[4] Ahmed H., Nandi A.K. (2020). Condition Monitoring with Vibration Signals—Compressive Sampling and Learning Algorithms for Rotating Machines.

[5] Parey A. (2016). A review of gear fault diagnosis using various condition indicators. Procedia Eng..

[6] (1995). Gears—Wear and Damage to Gear Teeth.

[7] Liu H., Liu H., Zhu C., Zhou Y. (2019). A Review on Micropitting Studies of Steel Gears. Coatings.

[8] Henriquez P., Alonso J.B., Ferrer M.A., Travieso C.M. (2014). Review of Automatic Fault Diagnosis Systems Using Audio and Vibration Signals. IEEE Trans. Syst. Man Cybern. Syst..

[9] Zakrajsek J., Townsend D.P., Decker H.J. (1993). An Analysis of Gear Fault Detection Methods as Applied to Pitting Fatigue Failure Data.

[10] Lupea I., Lupea M. (2023). Machine Learning Techniques for Multi-Fault Analysis and Detection on a Rotating Test Rig using Vibration Signal. Symmetry.

[11] Hu C., Smith W.A., Randall R.B., Peng Z. (2016). Development of a gear vibration indicator and its application in gear wear monitoring. Mech. Syst. Signal Process..

[12] Kim J., Kim J., Kim H.A. (2022). A Study on Gear Defect Detection via Frequency Analysis Based on DNN. Machines.

[13] Dennis G. (1946). Theory of Communications. J. Inst. Electr. Eng..

[14] Zappalà D., Tavner P.J., Crabtree C.J., Sheng S. (2013). Side-band Algorithm for Automatic Wind Turbine Gearbox Fault Detection and Diagnosis. IET Renew. Power Gener..

[15] Li H., Zhang Y., Zheng H. (2009). Gear fault detection and diagnosis under speed-up condition based on order cepstrum and radial basis function neural network. J. Mech. Sci. Technol..

[16] Yu D., Yang Y., Cheng J. (2007). Application of time–frequency entropy method based on Hilbert–Huang transform to gear fault diagnosis. Measurement.

[17] Yang R., Zhong M. (2022). Machine Learning-Based Fault Diagnosis for Industrial Engineering Systems.

[18] Zhao R., Yan R., Chen Z., Mao K., Wang P., Gao R.X. (2019). Deep learning and its applications to machine health monitoring. Mech. Syst. Signal Process..

[19] Wang P., Ananya, Yan R., Gao R.X. (2017). Virtualization and deep recognition for system fault classification. J. Manuf. Syst..

[20] Yu L., Yao X., Yang J., Li C. (2020). Gear Fault Diagnosis through Vibration and Acoustic Signal Combination Based on Convolutional Neural Network. Information.

[21] Kaplan K., Kaya Y., Kuncan M., Mlnaz M.R., Ertunc H.M. (2020). An improved feature extraction method using texture analysis with LBP for bearing fault diagnosis. Appl. Soft Comput..

[22] Li X., Li J., Qu Y., He D. (2019). Gear Pitting Fault Diagnosis Using Integrated CNN and GRU Network with Both Vibration and Acoustic Emission Signals. Appl. Sci..

[23] Grosse C.U., Reinhardt H.W., Finck F. (2003). Signal-Based Acoustic Emission Techniques in Civil Engineering. J. Mater. Civ. Eng..

[24] Plum R., Ummenhofer T. (2013). Use of Ultrasound Excited Thermography Applied to Massive Steel Components: Emerging Crack Detection Methodology. J. Bridge Eng..

[25] Qu Y., He M., Deutsch J., He D. (2017). Detection of Pitting in Gears Using a Deep Sparse Autoencoder Appl. Sci..

[26] Mohammed S.A., Ghazaly N.M., Abdo J. (2022). Fault Diagnosis of Crack on Gearbox Using Vibration-Based Approaches. Symmetry.

[27] Kiranyaz S., Avci O., Abdeljaber O., Ince T., Gabbouj M., Inman D.J. (2021). 1D convolutional neural networks and applications: A survey. Mech. Syst. Signal Process..

[28] Goodfellow I., Bengio Y., Courville A. (2017). Deep Learning.

[29] Brown L., Cai T., DasGupta A. (2001). Interval estimation for a binomial proportion. Stat. Sci..

